# Serum chitotriosidase and YKL-40 in acute pancreatitis: Reliability as prognostic marker for disease severity and correlation with inflammatory markers

**DOI:** 10.3906/sag-2106-59

**Published:** 2021-09-27

**Authors:** Ece ÜNAL ÇETİN, Fatih KAMIŞ, Adil Ugur ÇETİN, Yavuz BEYAZIT, Murat KEKİLLİ

**Affiliations:** 1 Department of Internal Medicine, Faculty of Medicine, Çanakkale Onsekiz Mart University, Çanakkale, Turkey; 2 Division of Gastroenterology, Department of Internal Medicine, Faculty of Medicine, Çanakkale Onsekiz Mart University, Çanakkale, Turkey; 3 Division of Gastroenterology, Department of Internal Medicine, Faculty of Medicine, Gazi University, Ankara, Turkey

**Keywords:** Acute pancreatitis, YKL-40, chitotriosidase, inflammation

## Abstract

**Background/aim:**

Chitotriosidase and YKL-40, also called chitinase 3-like protein 1, are homologs of family 18 glycosyl hydrolases, secreted by human macrophages and granulocytes under inflammatory conditions. Although increased levels of chitotriosidase and YKL-40 are linked with several inflammatory diseases, the physiological utility of these two enzymes is still not fully characterized. This study aims to analyse the serum YKL-40 and chitotriosidase levels of acute pancreatitis patients to assess whether their activity correlates with acute pancreatitis and its severity.

**Materials and methods:**

Chitotriosidase and YKL-40 levels, along with routine laboratory parameters, were determined from the serum samples of 41 acute pancreatitis patients, at both onset and remission (male/female: 22/19), and 39 healthy subjects (male/female: 19/20). The Modified Glasgow Prognostic Score was used to predict the severity of the disease, and a correlation analysis was performed between study variables.

**Results:**

A statistically significant increase in both chitotriosidase and YKL-40 levels was observed in acute pancreatitis patients compared to healthy controls (P < 0.001). Higher levels of YKL-40, chitotriosidase and C-reactive protein were found in patients with acute pancreatitis at onset than in remission. The correlation analysis showed a statistically significant association between YKL-40 and chitotriosidase (p = 0.039, r = 0.323). The cut-off point for YKL-40, for detecting acute pancreatitis, was 60.3 with a sensitivity and specificity of 84.9% and 84.6% (AUC: 0.890). The optimum cut-off points for chitotriosidase, for detecting acute pancreatitis, was 33.5 with a sensitivity and specificity of 79.5% and 78.4% (AUC: 0.899).

**Conclusion:**

Elevated YKL-40 and chitotriosidase levels in acute pancreatitis patients demonstrate the importance of possible macrophage involvement in the pancreatic microenvironment during acute pancreatitis progression.

## 1. Introduction

Acute pancreatitis (AP) is a serious but reversible inflammatory process of the pancreatic tissue that can progress to systemic inflammatory response syndrome with considerable morbidity and mortality in approximately one-fifth of patients [1]. A sudden onset of abdominal pain in conjunction with a rise of pancreatic enzymes that are released from the acinar cells of the pancreas are the basis for an AP diagnosis. Both amylase and lipase can be elevated in distinct disease conditions, including perforated gastric or duodenal ulcers, renal insufficiency, intestinal obstruction, tubo-ovarian disease, and mesenteric infarction [2]. Although less widely available in clinical conditions, more specific rapid supplemental diagnostic tests, such as urinary trypsinogen-2 and trypsinogen activation peptide, do exist [3,4]. Due to the potential for catastrophic deterioration, it is crucial to achieve diagnostic stratification of injury severity and diagnostic accuracy by the adjunctive use of additional diagnostic assays. In this context, multiple risk stratification scores, with different clinical and biochemical variables, are proposed to determine the severity of AP. These scores include the Ranson criteria, APACHE II score, SOFA, and modified Glasgow Prognostic score (mGPS) [5]. However, these systems are sometimes criticized as unnecessarily complex, requiring multiple measurements and difficult to perform for patients outside the intensive care unit due to too many parameters they use.

Chitin is a long-chain polysaccharide, which is the major constituent of bacteria cell walls, the sheaths of parasitic nematodes and fungi [6]. In mammals, despite lacking endogenous chitin, several chitinase-like proteins have been determined, though their function is not fully understood [7]. Humans and mice express two active chitinases, namely chitotriosidase (CHIT1) and acidic mammalian chitinase (AMCAse), and at least three chitinase-like proteins (CLPs), namely YKL-40 (chitinase 3-like protein 1), YKL-39 (chitinase 3-like protein 2) and oviductin [8].

Chitotriosidase is a part of the chitinase enzyme family and participates in the degradation of chitin and chitin-like substrates because of hydrolyses of N-acetyl-beta-glucosaminide(1–4)-beta linkages. With this functional property, chitotriosidase exerts antibacterial, fungicidal and parasiticidal effects and participates in immune defence. Chitotriosidase does so by involving macrophage plasticity, presenting nonchitin antigen-derived peptides and effecting the straight stimulation of many inflammatory cytokines, for instance interleukin-8 (IL-8) and tumour growth factor-beta (TGF-β) [9]. Although no chitotriosidase activity has been reported in the human metabolism, chitotriosidase has been proposed to play a key function in innate immunity and is considered a useful marker of macrophage activation [10].

YKL-40 is a 40 kDa heparin- and chitin-binding glycoprotein, which is secreted by various cells, including vascular smooth muscle cells and macrophages. As a novel inflammatory marker, YKL-40 has been found to be elevated in several inflammatory and neoplastic disease conditions, including atherosclerosis, benign prostatic hyperplasia, diabetes mellitus, osteoarthritis (OA), polymyositis, psoriasis, rheumatoid arthritis, cervical cancer, and primary prostate cancer [11–18]. Although Johansen et al. [19] found elevated serum YKL-40 levels in AP patients, they failed to explore the relationship between chitotriosidase and conventional markers of inflammation.

Based on the emerging roles of YKL-40 and chitotriosidase in diseases characterized by inflammation and fibrosis, we designed this study to explore the possible role of YKL-40 and chitotriosidase in AP and to determine whether these two parameters could be related to the disease activity. Meanwhile, we also compared the clinical significance of YKL-40 and chitotriosidase with systemic markers of inflammation.

## 2. Materials and methods

### 2.1. Study participants

This prospective study comprised 41 patients with biliary AP who were admitted to Çanakkale Onsekiz Mart University (COMU) Hospital between September 2019 and December 2020. The patients were followed during their hospitalization period, and the mean hospitalization time was 4.1 ± 3.2 days. AP diagnosis was established according to the presence of severe abdominal pain, vomiting and elevated serum amylase/lipase concentration (more than three times the upper normal limit) or characteristic radiological imaging, compatible with AP based on the Atlanta classification [20]. The clinical follow-up of each patient was performed prospectively until each patient was discharged from hospital. Remission was defined as symptom disappearance, normal pancreatic enzyme levels and starting oral nutrition. Exclusion criteria was defined as the existence of acute or chronic inflammatory disorders, peripheral vascular disease, tumoral conditions, renal and hepatic diseases, objective evidence, or prior diagnosis of chronic pancreatitis, or use of nonsteroidal antiinflammatory and anticoagulant drugs. Healthy controls were chosen from the university and hospital staff. All healthy controls were selected by an internal medicine specialist and presented no complaints, symptoms, or history of any tumoral, renal, hepatic, metabolic, or endocrinologic disease conditions.

This study was performed in accordance with the Declaration of Helsinki and approved by the COMU Institutional Ethics Board (Approval date: 11.12.2019; No: 20–05). Prior to study enrolment, signed informed consent was obtained from all the study participants.

### 2.2. Clinical and laboratory assessment

For each patient, the following clinical, laboratory and demographic data were recorded: age, sex, aetiology of AP (estimated by clinical history, physical examination, routine blood tests and radiologic evaluations) and disease severity (determined by a modified Glasgow prognostic score (mGPS) within 48 h of admission). Eight parameters of mGPS were evaluated, and the patients were graded as mild (score < 3) or severe (score ≥ 3) according to the total sum of the scores [21].

Routine complete blood count and biochemical parameters, including amylase, lipase, alanine aminotransferase, aspartate aminotransferase, albumin, calcium, glucose, lactate dehydrogenase, blood urea nitrogen, creatinine, erythrocyte sedimentation rate and C-reactive protein (CRP) were determined. Fasting blood samples were collected, at both the onset and remission of the disease, from the antecubital vein after overnight fasting without using any anticoagulant. Blood samples were left on the clot, and serum was separated from cellular elements by centrifugation (3000 rounds per minute for 15 min) within 2 h after blood sampling. All serum samples were stored at −80 °C until the analysis was performed.

### 2.3. YKL-40 and chitotriosidase assay

Serum YKL-40 levels were measured by a double antibody enzyme-linked immunosorbent assay (ELISA) kit, from Sunred Biotechnology Company, made in Shanghai, China, Catalogue Number 201-12-2144. Chitotriosidase concentrations were determined using commercially available ELISA kits (Sunred, Shanghai, China), Catalogue Number 201-12-3460.

### 2.4. Statistical analysis

All statistical analysis was performed using the SPSS statistical program for Windows (IBM SPSS Statistics, Version 20.0. Armonk, NY: IBM Corp). Continuous and categorical variables were presented as median interquartile range or mean ± standard deviation and n (%), respectively. The chi-square test was used to determine significant relationship between categorical variables. The Shapiro–Wilk test was used to determine whether study parameters had normal distribution in study groups, and the Mann–Whitney test or Student’s t-test was used to calculate the significance of the differences between the two groups. For multiple comparisons one-way ANOVA test, followed by Bonferroni post hoc test was performed and adjusted p values were calculated. The Spearman correlation analysis was used to identify the correlation between YKL-40 and chitotriosidase, with other markers of inflammation. Odds ratios (95% confidence intervals) of the independent clinical and laboratory parameters were calculated with univariate logistic regression model to predict AP. Receiver operating characteristic (ROC) curve analysis was used to identify optimal cut-off values of YKL-40, chitotriosidase and other inflammation markers with highest sensitivity and specificity for detection of AP severity.

## 3. Results

**Figure 1:**
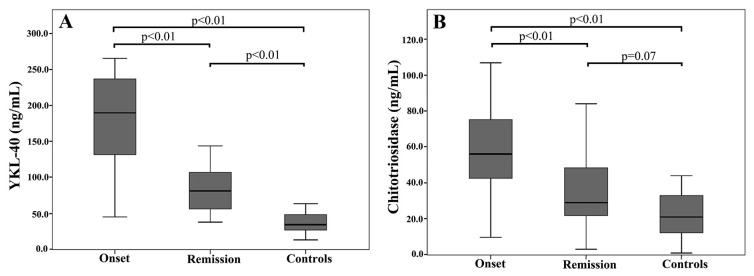
YKL-40 (A) and chitotriosidase (B) levels of the acute pancreatitis patients at onset and remission in comparison with healthy controls.

Forty-one patients with biliary AP and 39 healthy subjects were included in the present study. Twenty-two (53.6%) of the patients with AP and 19 (48.7%) of the healthy subjects were male. The median ages of the AP and control patients were 69 (37–89) years and 64 (23–88) years, respectively. No significant differences were observed in respect to the ages and genders of the study groups. The mean serum YKL-40 levels in the AP patients were 180.49 ± 62.01 ng/mL and was found to be significantly elevated (p < 0.001) compared with the level in the control group (36.12 ± 14.14). The mean serum chitotriosidase levels in the AP patients was 58.44 ± 24.43 ng/mL and was significantly elevated (p < 0.001) compared with the level in the healthy controls (21.48 ± 11.81). Figure 1 shows the mean YKL-40 (Figure 1A) and chitotriosidase (Figure 1B) levels of the AP patients at the onset and remission of AP compared with those of the healthy controls. The clinical characteristics and laboratory values, including conventional markers of inflammation, of the study groups are given in Table 1 .

**Figure 2:**
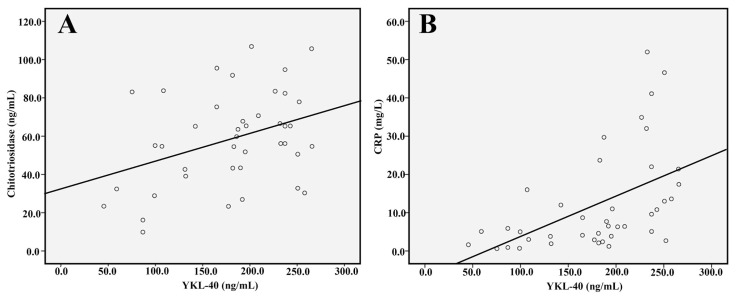
Comparison of YKL-40 with chitotriosidase (A) and C reactive protein (CRP) (B)

The comparison of serum YKL-40 and chitotriosidase levels and other systemic inflammation markers at onset and remission are shown in Table 2. Higher YKL-40, chitotriosidase, white blood cell (WBC), erythrocyte sedimentation rate and CRP were demonstrated in the AP patients at the onset of the disease compared with the remission. A correlation analysis revealed a significant correlation between YKL-40 and chitotriosidase (r = 0.323, p = 0.039) (Figure 2A). Although a significant correlation was found between CRP and YKL-40 (r = 0.591, p < 0.001) (Figure 2B), no significant correlation was observed between CRP and chitotriosidase (r = 0.172, p = 0.282). Table 3 summarizes the correlation analysis of study variables in AP patients at the onset of the disease.

In order to predict AP, we also created a univariable logistic regression model to study the associations between AP and independent inflammatory markers including YKL-40 and chitotriosidase (Table 4). We found a significant improvement in performance using conventional inflammatory markers as well as YKL-40 and chitotriosidase according to univariable regression analysis. Multivariable analysis was not performed because of the relatively low number of cases.

The severity of AP was calculated according to the mGPS. According to this scoring system, 22 patients (53.7%) were classiﬁed as having mild AP, and 19 patients (46.3%) were classified as having severe AP. According to the disease severity, Table 5 provides the comparison of YKL-40 and chitotriosidase with other study variables at the onset of the disease.

The Receiver Operating Characteristic (ROC) curve analysis suggested that the optimum YKL-40 level cut-off points for determining AP was ≥60.3 ng/mL with a sensitivity, specificity, negative predictive value (NPV) and positive predictive value (PPV) of 84.9%, 84.6%, 78.9% and 82.6, respectively (AUC: 0.890). Optimum chitotriosidase level cut-off points for determining AP was ≥33.5 ng/mL with a sensitivity, specificity, NPV and PPV of 79.5%, 78.4%, 79.3% and 78.6, respectively (AUC: 0.899) (Table 6). YKL-40 and chitotriosidase failed to demonstrate a certain cut-off value, with adequate sensitivity and specificity, for differentiating mild AP patients from severe AP patients (Table 6).

## 4. Discussion

This study has shown that YKL-40 and chitotriosidase levels are elevated in patients with AP in comparison to in healthy controls. Both enzyme levels were found to be decreased after treatment. Interestingly, the YKL-40 and chitotriosidase activity was not affected by the initial severity of the disease, suggesting that some factor or factors common to all stages of the disease, apart from inflammation, may play a significant role in the course of the disease. Furthermore, serum YKL-40 and chitotriosidase levels were found to have high sensitivity, specificity, and predictive values in patients with AP. Based on the data we provided, it can be suggested that both YKL-40 and chitotriosidase may be regarded as valuable biomarkers of inflammation in AP patients. Thus, our findings add new and relevant proof to the growing body of literature on the role of chitinases and chitin-like proteins in the physiopathology of AP.

As the incidence of AP is increasing, it is imperative to determine the severity of the disease to recognize patients that are at risk of developing highly unfavourable outcomes. To achieve these goals, the determination of inflammatory activity has considerable prognostic relevance. Apart from clinical assessments, biochemical and radiological evaluations are commonly used to determine the presence and the severity of pancreatic inflammation [2,22]. Unfortunately, determining AP severity in the early phases of the disease is still challenging and depends chiefly on analyzing different clinical and biochemical variables. Therefore, there is an eager demand for a simple and inexpensive method that can precisely evaluate the presence and the severity of AP. In this context, there are several biochemical assays that are being used routinely or on an as-needed basis in AP diagnosis. However, to the best of our knowledge, no previous studies have been undertaken to simultaneously evaluate YKL-40 and chitotriosidase in the peripheral blood of patients with AP.

Chitotriosidase belongs to a family of various chitinase and chitinase-like proteins and is involved in defence against chitin-containing pathogens [23]. Chitotriosidase is mainly produced by activated macrophages and granulocytes under inflammatory conditions. As is well known, macrophages in the pancreas and other associated organs are mostly activated in AP and, furthermore, secrete inflammatory cytokines and mediators, which determine the severity of pancreatitis [24]. However, elevated chitotriosidase secretion in acute and chronic inflammatory disease conditions, including lysosomal storage diseases, sarcoidosis, interstitial lung disease, tuberculosis, acute malaria (induced by Plasmodium falciparum) and granulomatous diseases have been reported; to the best of our knowledge, this is the first study showing the role of chitotriosidase in AP patients [10,25–27]. The results of the present study have demonstrated elevated levels of chitotriosidase in AP patients, suggesting a prompt activation of pancreatic macrophages in the disease course regardless of the disease severity.

This study revealed increased YKL-40 levels in AP patients irrespective of disease severity, as reflected by mGPS. YKL-40 and chitotriosidase belong to the same family, “mammalian chitinase-like proteins,” but YKL-40 is not a chitinase; rather, it binds tightly to chitin particles [28]. YKL-40 is a potential serum biomarker of distinct benign and malignant disease states, including diabetes mellitus, coronary artery disease, systemic sclerosis, psoriasis, dementia, benign prostatic hyperplasia, cervical cancer, and primary prostate cancer [14–17, 28, 29]. Unfortunately, there is scarce evidence for an association between YKL-40 and AP. In this context, the Johansen et al.’s [19] study is the first and only study in the literature that demonstrates the role of YKL-40 in patients with AP. The authors not only reported elevated serum YKL-40 levels in AP patients at admission but also much higher serum YKL-40 levels in patients with severe disease than mild disease. Apart from AP, the association between YKL-40 and pancreatic cancer has recently been demonstrated in a large series of patients with unresectable pancreatic cancer [30]. Serum YKL-40, IL6, and CRP were found to be elevated in advanced-stage cancer patients with poor performance statuses. Moreover, combined elevations of YKL-40, serum IL6, and CRP were reported to be associated with worse survival rates in contrast to isolated, high concentrations in a single marker. Although we have demonstrated a significant increase in YKL-40 levels in AP patients, we did not observe a significant difference regarding disease severity in respect to YKL-40 levels in AP patients. Based on these data, we suggest that YKL-40 plays a pivotal role in AP, irrespective of the severity of the disease, possibly by regulating key pathways and processes within the respective pancreatic microenvironment during AP. These processes include inflammation, angiogenesis, cell proliferation, differentiation, and remodelling of the extracellular matrix.

Although there is no ideal single serum marker for predicting the presence and the severity of AP, CRP is a practical marker of inflammation and necrosis, with a specificity and sensitivity of 80%. However, CRP must be measured more than 48 h after the beginning of clinical symptoms because the peak CRP level appears 24 to 48 ho after the onset of pancreatitis [2, 31, 32]. Regarded as an acute phase reactant, circulating YKL-40 may provide novel information regarding the severity of inflammatory disease, as has already been demonstrated by multiple studies [33, 34]. In patients with endotoxemia, which is followed by increased plasma IL-6 and TNF-alpha levels, plasma YKL-40 increased earlier than serum CRP, which is a nonspecific acute-phase protein synthesized in the liver in response to stimulation, mainly from IL6 [19, 35]. YKL-40 is secreted rapidly by activated monocytes and neutrophils because of ongoing inflammatory response, and this premature elevation of YKL-40 from the CRP makes it a suitable candidate for AP diagnosis [19, 36]. This study has revealed a positive correlation between serum CRP and YKL-40 levels, but no association was found between CRP and chitotriosidase levels. Similarly, Johansen et al. [19] found a positive correlation between serum YKL-40 and CRP levels at admission and after 48 h (admission: r = 0.70; 48 h: r = 0.66) (p <0.0001).

The primary limitation of this study is the relatively small sample size used for the analyses. Additionally, based on the acute inflammatory nature of AP, it would have enhanced our study if we had simultaneously measured other well-known inflammatory factors that play vital roles in the pathological process of AP, such as IL-6, IL-8, TNF-alpha. Yet another limitation of this study was that the duration of patient follow-up was very short, and it would have been beneficial to monitor the changes in YKL-40 and chitotriosidase over a longer period.

## 5. Conclusion

Despite the above-mentioned limitations of the current study, we have demonstrated for the first time that YKL-40 and chitotriosidase are simple, reliable, and inexpensive potential biomarkers of AP, with high specificity and sensitivity rates. Moreover, we propose that elevated serum YKL-40 and chitotriosidase activity in patients with AP suggests the importance of possible macrophage involvement in the pancreatic microenvironment during AP progression. Both assays may be combined with other markers of inflammation to detect the presence and severity of the disease and to fortify the efficacy of the treatment.

## Figures and Tables

**Table 1:** Demographic and laboratory data of the patients with acute pancreatitis and healthy controls.

	Acute pancreatitis (n = 41)	Control group (n = 39)	t, z, or Chi-square	p
Age (years)	69 (37–90)	64 (23–80)	−1.050^a^	0.201
Gender (F/M)	19/22	20/19	0.195^b^	0.823
WBC (/mm^3^ × 10^3^)	12.80 ± 6.23	7.37 ± 2.79	5.071^c^	< 0.001
Hemoglobin (g/dL)	12.9 ± 2.22	12.62 ± 1.95	0.732^c^	0.467
Platelet (/mm^3^ × 10^3^)	257.49 ± 83.09	245.26 ± 113.33	0.552^c^	0.582
NLR	12.05 ± 2.07	2.77 ± 1.73	3.564^c^	<0.001
PLR	268.02 ± 191.64	139.25 ± 62.85	4.435^c^	<0.001
CRP (mg/L)	12.19 ± 13.19	0.72 ± 0.95	5.552^c^	< 0.001
Sedimentation (mm/h)	37.00 ± 25.45	19.62 ± 12.45	3.750^c^	< 0.001
YKL-40 (ng/mL)	180.49 ± 62.01	36.12 ± 14.14	14.514^c^	< 0.001
Chitotriosidase (ng/mL)	58.44 ± 24.43	21.48 ± 11.81	8.678^c^	< 0.001

a nonparametric test;

b chi-square test;

c independent sample t-test;

WBC: White blood cell, NLR: Neutrophil-lymphocyte ratio, PLR: Platelet-lymphocyte ratio, CRP: C-reactive protein.

**Table 2:** Comparison of serum YKL-40 and chitotriosidase levels and other markers of inflammation at onset and remission of acute pancreatitis patients (n = 41).

	Onset	Remission	t-value	p
WBC (/mm^3^×10^3^)	12.80 ± 6.23	6.98 ± 2.20	5.641	<0.001
Platelet (/mm^3^×10^3^)	257.49 ± 83.09	267.98 ± 116.75	−0.469	0.882
NLR	12.05 ± 2.07	3.48 ± 1.74	3.480	<0.001
PLR	268.02 ± 191.64	201.56 ± 132.77	0.967	0.044
Sedimentation (mm/h)	37.00 ± 25.45	20.68 ± 12.88	3.508	0.005
CRP (mg/dL)	12.19 ± 13.19	1.20 ± 1.01	5.320	<0.001
YKL-40 (ng/mL)	180.49 ± 62.01	85.50 ± 32.78	8.671	<0.001
Chitotriosidase (ng/mL)	58.44 ± 24.43	34.49 ± 17.71	5.082	<0.001

WBC: White blood cell, NLR: Neutrophil-lymphocyte ratio, PLR: Platelet-lymphocyte ratio, CRP: C-reactive protein.

**Table 3:** Correlation analysis of study variables at onset of acute pancreatitis.

	Chitotriosidase		YKL-40		Sedimentation		CRP		WBC	
	r	p	r	p	r	p	r	p	r	p
WBC	0.106	0.509	−0.244	0.123	0.316	0.057	−0.341	0.029	-	-
CRP	0.172	0.282	0.591	<0.001	0.055	0.746	-	-	-	-
Sedimentation	−0.109	0.520	−0.258	0.124	-	-	-	-	-	-
YKL-40	0.323	0.039	-	-	-	-	-	-	-	-
Chitotriosidase	-	-	-	-	-	-	-	-	-	-

WBC: White blood cell, CRP: C-reactive protein.

**Table 4:** Univariable logistic regression analysis to determine acute pancreatitis in patients that admitted to emergency department.

	Univariable analysis		
	p value	OR	(95% C.I.)
Age	0.201	1.020	0.989–1.053
Sex	0.659	1.219	0.507–2.933
WBC	<0.001	1.482	1.219–1.802
CRP	<0.001	3.340	1.823–6.119
Sedimentation	0.001	1.050	1.019–1.081
YKL-40	0.011	1.163	1.036–1.308
Chitotriosidase	<0.001	1.120	1.065–1.178

**Table 5:** Comparison of YKL-40 and chitotriosidase with other study variables at onset of acute pancreatitis (AP) according to disease severity measured by modified Glasgow Prognostic score.

	Mild AP	Severe AP	t-value	p
WBC (/mm^3^×10^3^)	13.20 ± 7.57	12.34 ± 4.36	0.432	0.865
Platelet (/mm^3^×10^3^)	247.95 ± 84.82	268.52 ± 81.91	0.787	0.456
NLR	11.28 ± 10.51	12.95 ± 11.10	0.658	0.583
PLR	241.16 ± 186.64	299.14 ± 197.67	0.786	0.360
Sedimentation(mm/h)	27.63 ± 19.03	46.88 ± 28.05	−2.455	0.023
CRP (mg/dL)	7.83 ± 8.76	17.24 ± 15.72	−2.410	0.038
YKL-40 (ng/mL)	186.32 ± 58.39	173.72 ± 66.95	−0.553	0.676
Chitotriosidase (ng/mL)	60.98 ± 26.55	55.48 ± 22.05	0.724	0.464

WBC: White blood cell, NLR: Neutrophil-lymphocyte ratio, PLR: Platelet-lymphocyte ratio, CRP: C-reactive protein.

**Table 6:** Overall accuracy and ROC analyses of YKL-40 and chitotriosidase with other conventional inflammation markers to determine acute pancreatitis and differentiate mild cases from severe cases according to the modified Glasgow Prognostic score.

Acute pancreatitis vs. controls	AUC	Cut-Off	Sensitivity (%)	Specificity (%)	PPV (%)	NPV (%)
WBC	0.827	8.45	79.5	81.1	80.8	79.8
NLR	0.874	4.1	89.2	81.4	80.5	89.7
PLR	0.785	170.0	73.8	73.7	75.6	71.8
Platelet	0.563	236.5	56.4	56.8	56.6	56.6
CRP	0.959	1.46	89.7	89.2	89.3	89.6
Sedimentation	0.699	20.0	69.2	70.3	70.0	69.5
YKL-40	0.890	60.3	84.9	84.6	82.6	78.9
Chitotriosidase	0.899	33.5	79.5	78.4	78.6	79.3
**Mild vs severe acute pancreatitis**	AUC	Cut-Off	Sensitivity (%)	Specificity (%)	PPV (%)	NPV (%)
WBC	0.545	8.85	54.1	55.0	54.6	54.5
NLR	0.602	7.1	57.9	50.0	50.0	57.9
PLR	0.626	202.0	63.2	50.0	52.2	61.1
Platelet	0.571	244	54.1	55.0	54.6	54.5
CRP	0.570	2.05	51.4	52.5	52.0	51.9
Sedimentation	0.692	23.5	62.2	65.0	64.0	63.2
YKL-40	0.580	107.77	59.5	57.5	58.3	58.7
Chitotriosidase	0.474	42.03	48.6	47.5	48.1	48.0

WBC: White blood cell, NLR: Neutrophil-lymphocyte ratio, PLR: Platelet-lymphocyte ratio, CRP: C-reactive protein.
